# *Platycodon grandiflorus* Root Extract Attenuates Body Fat Mass, Hepatic Steatosis and Insulin Resistance through the Interplay between the Liver and Adipose Tissue

**DOI:** 10.3390/nu8090532

**Published:** 2016-08-30

**Authors:** Ye Jin Kim, Ji-Young Choi, Ri Ryu, Jeonghyeon Lee, Su-Jung Cho, Eun-Young Kwon, Mi-Kyung Lee, Kwang-Hyeon Liu, Yu Rina, Mi-Kyung Sung, Myung-Sook Choi

**Affiliations:** 1 Center for Food and Nutritional Genomics Research, Kyungpook National University, 1370 San-Kyuk Dong Puk-Ku, Daegu 41566, Korea; freewilly59@hanmail.net (Y.J.K.); jyjy31@hanmail.net (J.-Y.C.); sangsang0119@gmail.com (R.R.); wjdgus4411@naver.com (J.L.); chocrystalhihi@hanmail.net (S.-J.C.); savagegarden01@hanmail.net (E.-Y.G); 2 Department of Food Science and Nutrition, Kyungpook National University, 1370 San-Kyuk Dong Puk-Ku, Daegu 702-701, Korea; 3 Department of Food and Nutrition, Sunchon National University, Suncheon 540-950, Korea; leemk@sunchon.ac.kr; 4 College of Pharmacy and Research Institute of Pharmaceutical Sciences, Kyungpook National University, 1370 San-Kyuk Dong Puk-Ku, Daegu 702-701, Korea; dstlkh@gmail.com; 5 Department of Food Science and Nutrition, University of Ulsan, Ulsan 44610, Korea; rinayu@ulsan.ac.kr; 6 Department of Food and Nutrition, Sookmyung Women’s University, Seoul 04310, Korea; mksung@sookmyung.ac.kr

**Keywords:** *Platycodon grandiflorus* root, obesity, free fatty acid oxidation, energy expenditure, thermogenesis

## Abstract

The *Platycodon grandiflorus* root, a Korean medicinal food, is well known to have beneficial effects on obesity and diabetes. In this study, we demonstrated the metabolic effects of *P. grandiflorus* root ethanol extract (PGE), which is rich in platycodins, on diet-induced obesity. C57BL/6J mice (four-week-old males) were fed a normal diet (16.58% of kilocalories from fat), high-fat diet (HFD, 60% of kilocalories from fat), and HFD supplemented with 5% (*w*/*w*) PGE. In the HFD-fed mice, PGE markedly suppressed the body weight gain and white fat mass to normal control level, with simultaneous increase in the expression of thermogenic genes (such as *SIRT1*, *PPAR*α, *PGC1*α, and *UCP1*), that accompanied changes in fatty acid oxidation (FAO) and energy expenditure. In addition, PGE improved insulin sensitivity through activation of the PPARγ expression, which upregulates adiponectin while decreasing leptin gene expression in adipocytes. Furthermore, PGE improved hepatic steatosis by suppressing hepatic lipogenesis while increasing expression of FAO-associated genes such as *PGC1*α. PGE normalized body fat and body weight, which is likely associated with the increased energy expenditure and thermogenic gene expression. PGE can protect from HFD-induced insulin resistance, and hepatic steatosis by controlling lipid and glucose metabolism.

## 1. Introduction

Obesity is defined as excessive fat accumulation that may have adverse effects on health, such as type 2 diabetes, insulin resistance, atherosclerosis, dyslipidemia, hepatic steatosis, and cancer [1,2]. Elevated triglycerides, blood pressure, and fasting glucose levels, as well as reduced HDL cholesterol levels, are recognized as major risk factors for these disorders [3]. Obesity-induced inflammation, a key feature of adipose tissue dysfunction, has been proposed to be an important link between obesity and insulin resistance [4]. Adipose tissue is a critical component of metabolic control and an endocrine organ that secretes a number of adipokines, known to mediate lipid metabolism, inflammation, and insulin sensitivity [5].

Dysregulation of lipid metabolism in the liver induces abnormal accumulation of lipids and subsequent formation of lipid droplets (LDs), known as hepatic steatosis, which is common in obese individuals and strongly linked to insulin resistance [6]. Current strategies to ameliorate this disease include increasing energy expenditure and browning of white adipose tissue (WAT). In particular, genes involved in browning of WAT are associated with resistance to obesity, metabolic dysfunction, and hepatic insulin resistance [7,8].

The *Platycodon grandiflorus* root has long been used as a traditional medicine for treating bronchitis, asthma, pulmonary tuberculosis, hyperlipidemia, inflammation, and diabetes in Asia [9]. Recently, saponins from the root of *P. grandiflorus* showed a novel pharmacological potential as a treatment for metabolic diseases such as hyperlipidemia and diabetes [10]. The *P. grandiflorus* root, an oriental medicinal herb used in this study, has been approved in Korea as a food ingredient and used in oriental medicine for the treatment of obesity [11], although the mechanism of action of its extract has not been reported yet.

We investigated whether *P. grandiflorus* root ethanol extract (PGE) could attenuate adiposity, insulin resistance, hepatic steatosis, and inflammation in C57BL/6J mice fed a high-fat diet (HFD), a widely used animal model for obesity [12]. We also attempted to elucidate the molecular mechanism of PGE action by examining its effects on mRNA expression of browning markers in WAT of the diet-induced obesity (DIO) mice.

## 2. Materials and Methods

### 2.1. Preparation of Platycodon grandiflorus Root Extract

*P. grandiflorus* roots were obtained from Omniherb, Korea, and extracted with 10 volumes of 70% ethanol for 6 h at 50 °C. The extract (PGE) was collected and concentrated using a vacuum evaporator and then freeze-dried (yield 13.1%) for use in this study. The PGE contained bioactive components such as triterpenoid saponins as determined by liquid chromatography–mass spectrometry (Table S1).

### 2.2. Animals and Diets

Male C57BL/6J mice (4-week-old) were obtained from The Jackson Laboratory (Bar Harbor, ME, USA). All mice were individually housed under a constant temperature (24 °C) and 12-h light/dark cycle, fed a normal chow diet for a one-week acclimation period, and subsequently randomly divided into three groups. The mice in the different groups were fed, respectively, a normal diet (ND, 16.58% of kilocalories from fat, *n* = 10), HFD (60% of kilocalories from fat, *n* = 10), or HFD with 5% (*w*/*w*) of PGE (*n* = 10) for 12 weeks. The experimental diets were prepared every week and stored at 4 °C. At the end of the experimental period, all mice were anesthetized with ether after 12 h of fasting. Blood was taken from the inferior vena cava to determine the plasma lipid, adipokine, and hormone concentrations. The liver and adipose tissue were removed, rinsed with physiological saline, weighed, immediately frozen in liquid nitrogen, and stored at −70 °C until analysis. The animal study protocols were approved by the Ethics Committee at Kyungpook National University (Approval No. KNU 2015-0020).

Energy expenditure, morphology of the liver and fat tissues, glucose metabolism markers, hepatic lipid content, glucose- and lipid-regulating enzyme activity, and analysis of gene expression were performed as indicated in supporting information Material and Methods.

## 3. Results

### 3.1. Composition of Platycosides in PGE

Platycoside compounds of PGE were analyzed using LC-MS/MS method. The 18 platycoside compounds (about 4%) were identified in the PGE, which are deapioplatycoside E, platycoside E, deapioplatycodin D3, platycodin D3, platyconic acid B lactone, polygalacin D3, paltycoinc acid A, 3″-*O*-acetylplatyconic acid A, platycodin D2, platycodin D, 3″-*O*-acetylplatycodin D2, polygalacin D2, polygalacin D, 3″-*O*-acetylplatycodin D, platycodin V, platycodin A, 2″-*O*-actylpolygalacin D2, and 2″-*O*-actylpolygalacin D (Table S1). The most abundant platycoside compounds were platycodin A (10.28 μg/mg), 3″-*O*-acetylplatycodin D2 (10.25 μg/mg), 3″-*O*-acetylplatyconic acid A (3.50 μg/mg), 2″-*O*-actylpolygalacin D2 (2.34 μg/mg), and platycodin D3 (1.87 μg/mg).

### 3.2. PGE Supplement Lowered Body Weight Gain and Improved Plasma Lipid Profiles and Adipokine Levels in DIO Mice

**Figure 1:**
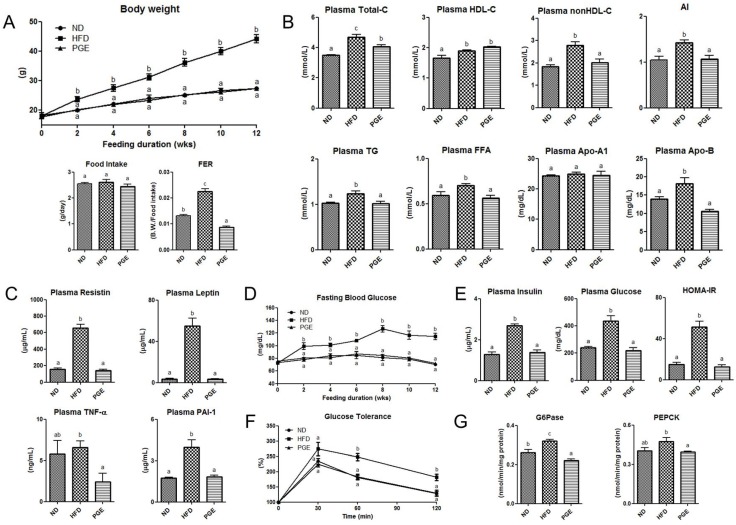
Effects of dietary PGE on body weight gain, plasma lipid profiles, insulin resistance, and glucose tolerance via modulating hepatic glucose-regulating enzyme activities in HFD-fed C57BL/6J mice. The data are the mean ± SEM (*n* = 10). (**A**) Changes in the body weight, food intake, and food efficiency ratio (FER); (**B**) levels of plasma total, HDL, and non-HDL cholesterol, AI, TG, FFA, Apo A-1, and Apo B; (**C**) levels of plasma resistin, leptin, TNF-α, and PAI-1; (**D**) blood glucose levels after 12 h of fasting; (**E**) plasma insulin and glucose levels after 12 h of fasting and the HOMA-IR calculated using the fasting blood glucose and insulin levels; (**F**) glucose tolerance; and (**G**) activities of the glucose-regulating enzymes G6Pase and PEPCK. ND, mice fed a normal diet; HFD, mice fed a high-fat diet (HFD) alone; PGE, *Platycodon grandiflorus* root extract (5%, *w*/*w*)-treated HFD-fed mice. B.W., body weight, AI, atherogenic index, ((Total-C)-(HDL-C))/HDL-C.

PGE significantly suppressed the body weight gain from the first week of high-fat feeding and decreased the food efficiency ratio, with no difference in food intake (Figure 1A). Interestingly, the addition of PGE led to a significant reduction of body weight compared to that in the HFD group, bringing it to the level similar to that in the ND group. PGE significantly decreased not only the total cholesterol (TC) and non-HDL cholesterol levels but also the apolipoprotein (Apo) B levels compared to those in the HFD group. The levels of plasma triglycerides (TG), and FFA were also markedly decreased by the PGE treatment compared to those in the HFD group (Figure 1B). Similar to the results for plasma lipid profiles, PGE significantly lowered the plasma adipokine levels, such as resistin, leptin, TNF-α, and plasminogen activator inhibitor-1 (PAI-1) levels (Figure 1C).

### 3.3. PGE Improved Insulin Resistance and Glucose Tolerance by Modulating Activities of Hepatic Glucose-Regulating Enzymes in DIO Mice

The fasting blood glucose concentration was significantly lowered by the PGE treatment after two weeks of high-fat feeding (Figure 1D). PGE significantly decreased the plasma insulin level as well as the HOMA-IR (Figure 1E). Moreover, the IPGTT revealed that PGE significantly improved glucose tolerance (Figure 1F), indicating a decrease in insulin resistance. Hepatic PEPCK and G6Pse activities were suppressed by the PGE treatment (Figure 1G).

### 3.4. PGE Supplement Decreased Body Fat Mass by Increasing Fatty Acid Oxidation-Related Gene Expression and Energy Expenditure in DIO Mice

**Figure 2:**
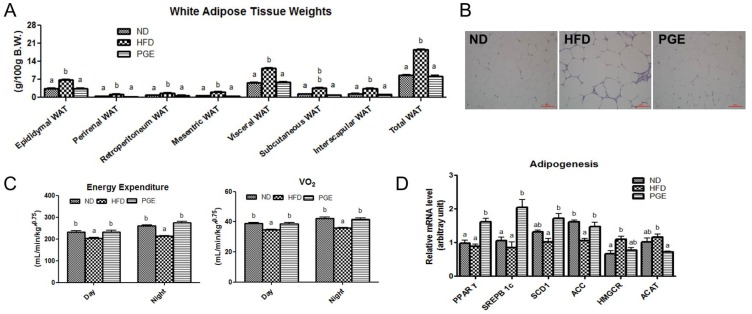

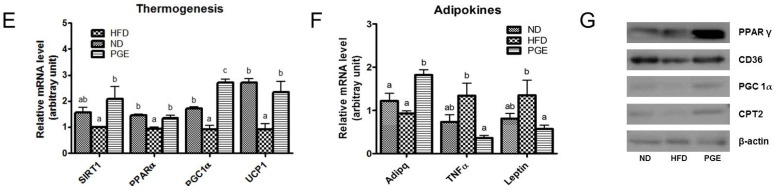
Effects of PGE on body fat mass, energy expenditure, and mRNA expression of adipogenic and thermogenic genes via regulation of fatty acid oxidation in epididymal white adipose tissue in HFD-fed C57BL/6J mice. The data are the mean ± SEM (*n* = 10). (**A**) The weight of adipose tissue; (**B**) representative photographs of adipocytes in the epididymal WAT of the mice, 200× magnification; and (**C**) energy expenditure and oxygen consumption (*V*o_2_). Adipogenesis (**D**); thermogenesis (**E**); and adipokine (**F**) related gene expression. (**G**) Western blot analysis of β-actin, PPARγ, CD36, *PGC1*α, and CPT2 expression. ND, mice fed a normal diet; HFD, mice fed a high-fat diet (HFD) alone; PGE, *Platycodon grandiflorus* root extract (5%, *w*/*w*)-treated HFD-fed mice; WAT, white adipose tissue.

The PGE-treated mice showed significantly upregulated mRNA expression levels for a key lipogenic gene, PPARγ, with simultaneous increases in mRNA expression of sterol regulatory element-binding protein 1c (SREBP-1c), stearoyl-CoA desaturase-1 (SCD1), and acetyl-CoA carboxylase 1 (ACC1) in eWAT (Figure 2D). However, similar to the results for body weight, PGE significantly reduced the weight of all WAT depots (including epididymal, perirenal, retroperitoneal, mesenteric, subcutaneous, and interscapular depots), with a decrease in the epididymal adipocyte size compared with the data obtained for the HFD group of the DIO mice (Figure 2A,B). In addition, whole-body oxygen consumption and energy expenditure of PGE-treat mice were significantly increased relative to those of HFD of the DIO mice (Figure 2C). Notably, PGE markedly reduced the weight of all adipose tissue depots to the level of that in the ND group. Importantly, the body fat reduction by the PGE treatment was associated with a significant increase in the expression of thermogenic genes, including the *SIRT1*, *PPAR*α, *PGC1*α, and *UCP1* genes (Figure 2E). Additionally, the PGE supplementation led to a significant increase in mRNA expression of adiponectin, with a decrease in the TNFα and leptin mRNA expression (Figure 2F). Western blot analysis revealed that the protein expression of the lipogenic and FA uptake factors, such as PPARγ and CD36, as well as FA oxidation related protein factors *PGC1*α and CPT2 (carnitine palmitoyltransferase 2) were markedly increased in the PGE-fed mice when compared with their expression in the eWAT of the HFD mice (Figure 2G).

### 3.5. PGE Supplement Lowered the Levels of Hepatic Lipids and Lipotoxicity Markers by Altering Hepatic Lipogenic Gene Expression and Enzyme Activities in DIO Mice

**Figure 3:**
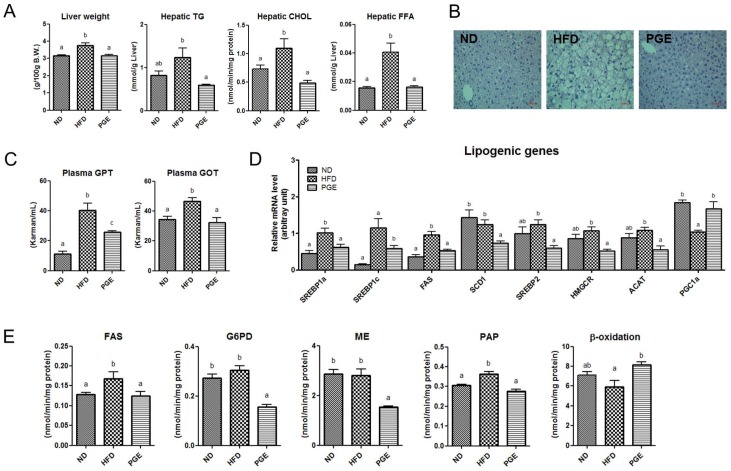
Effects of PGE treatment on hepatic steatosis-related markers in HFD-fed C57BL/6J mice. The data are the mean ± SEM (*n* = 10). (**A**) The weight of the liver and plasma levels of hepatic cholesterol, TG, and FFA; (**B**) hematoxylin and eosin (H & E)-stained transverse sections of the liver, 1000× magnification; (**C**) levels of the hepatic lipotoxicity markers GOT and GPT; (**D**) Lipogenesis-related gene expression; and (**E**) Hepatic lipid-regulating enzyme activities in the HFD-fed mice. ND, mice fed a normal diet; HFD, mice fed a high-fat diet (HFD) alone; PGE, *Platycodon grandiflorus* root extract (5%, *w*/*w*)-treated HFD-fed mice.

The PGE treatment markedly decreased the hepatic cholesterol, TG, and FFA content, as well as glutamic-oxaloacetic transaminase (GOT) and glutamic-pyruvic transaminase (GPT) levels in plasma (Figure 3A,C). In addition, PGE significantly decreased the mRNA expression of the genes and transcription factors involved in lipogenesis and cholesterol synthesis, with a simultaneous increase in FAO in the liver of the DIO mice (Figure 3D). Tissue morphology analysis also revealed that the accumulation of hepatic lipids was dropped and the cell size was decreased in the PGE group compared with those in the HFD group (Figure 3B). Furthermore, activities of the hepatic enzymes involved in FA and TG synthesis (FAS, G6PD, ME, and PAP) were significantly decreased by the PGE treatment, with a significant increase in the activity of β-oxidation in the liver (Figure 3E) compared with that in the HFD group.

## 4. Discussion

### 4.1. PGE Normalized Body Weight Gain and Fat Mass and Increased Expression of Browning Markers in eWAT

Brown adipose tissue (BAT) is a specialized tissue that dissipates energy in the form of heat (nonshivering thermogenesis) by uncoupling FAO from the ATP production via uncoupling protein 1 (*UCP1*) in mitochondria to protect against obesity [13]. In contrast, WAT does not normally express *UCP1* and is the main storage site of excess energy, primarily in the form of triglycerides, via the uptake of lipogenic substrates from the diet and de novo lipogenesis. Abnormal regulation of adipocyte differentiation, as well as lipogenesis, is linked to obesity [14]. Recently, it has been reported that in the obese, white adipocytes, known as “brite/beige” adipocytes (WAT cells that acquire a brown adipose phenotype, i.e., browning), can exhibit brown adipocyte-like characteristics by increasing expression of brown (thermogenic) genes, such as *SIRT1*, *UCP1*, *PGC1*α, and *PPAR*α [7,8]. An increase in the abundance of brite/beige adipocytes in WAT has been linked to the resistance to diet-induced obesity, with improved insulin resistance and increased energy expenditure, similar to the effects provided by the antidiabetic drug thiazolidinedione [15].

*P. grandiflorus* root (PG) has long been used as a traditional medicine and as a food in Asia. The major bioactivities components of PG are triterpenoid saponins (platycosides), such as platycodin A, D, and E, and platyconic acid, that may act individually, or in synergy to improve human health, including anti-obesity effects, anti-hyperlipidemia, and anti-inflammation [9,10,11].

In this study, the PGE treatment led to elevated lipogenesis through upregulation of PPARγ, thereby increasing the FA synthesis-associated gene expression (*SREBP1c*, *SCD1*, and *ACC1*), as well as increased FA uptake protein CD36 expression in eWAT. Thus, PGE led to increased FA re-esterification into newly synthesized TG by enhancing the adipose FA uptake and *SREBP1c*, *SCD1*, and *ACC1* genes. Interestingly, despite the activation of the lipogenic genes involved in FA synthesis in eWAT, the PGE supplement markedly decreased the weights of all WAT depots, as well as the body weight. This effect seemed to be associated with the increase in the mRNA expression of *SIRT1*, which is involved in thermogenesis and in the enhancement of the mRNA expression of FAO-associated genes genes and protein (*PPAR*α and *PGC1*α). Our data showed the upregulation of *SIRT1*, *PPAR*α gene expression, as well as increased *PGC1*α gene/protein expression by the PGE treatment. In addition, PGE supplementation increased expression of fatty acid oxidation related protein in eWAT, such as CPT2, compared to other HFD group. In rodents, activation of *SIRT1* in WAT promotes FAO by increasing the levels of *PPAR*α and its coactivator, *PGC1*α [8,16]. *PGC1*α is an important transcriptional coactivator for the expression of the *UCP1* gene, biogenesis of mitochondria, and energy expenditure in WAT [17]. Downregulation of *PGC1*α is associated with obesity and an increased risk of diabetes mellitus in the human population [18]. Additionally, obese animals treated with *PPAR*α agonists can benefit from thermogenesis induction, as the *PGC1*α-dependent myokine irisin which act through *PPAR*α to activate *UCP1* [19]. *UCP1*-mediated thermogenesis in WAT plays an important role in the regulation of energy expenditure [13]. Furthermore, *UCP1* is a major determinant of WAT thermogenic activity. Therefore, the increase of energy expenditure in the PGE-treated mice, associated with a significant increase in the mRNA expression of *UCP1*, can lead to the browning of WAT, which promotes thermogenesis and energy expenditure. In the obese, excessive lipid storage in WAT has been considered a key reason for obesity-associated insulin resistance and hepatosteatosis [20]. Taken together, it is plausible that PGE can contribute to browning of WAT in DIO mice via inducing FOA related genes (*SIRT1*, *PPAR*α, *PGC1*α, and *UCP1*) and proteins (PGC1α, and CPT2). Therefore, PGE can inhibit lipid accumulation via activation of FA oxidation in eWAT, despite increased of lipogenesis.

### 4.2. PGE Lowered Inflammatory Adipokines and Improved Insulin Resistance and Hepatic Steatosis by Alteration of Lipogenic Gene Expression and Glucose Metabolism

Furthermore, activation of PPARγ regulates the expression of adipocyte-secreted transcriptional factors such as adiponectin and leptin in WAT, which act as insulin sensitizers by potentiating insulin signaling in adipocytes [21,22]. Therefore, high adiponectin and/or low leptin levels can enhance insulin sensitivity in WAT and increase FAO in the liver, thus leading to the improvement of diabetes [15].

In our study, the increase in insulin sensitivity by the PGE treatment could also be related to PPARγ activation that led to an increase in adiponectin mRNA expression and a decrease in that of leptin in WAT. Elevation of adiponectin levels was correlated with a reduction of the hepatic fat content [23], similar to the effect of the PGE treatment. The hepatic TG and TC levels were significantly decreased compared with those in the DIO mice, which was accompanied by increased expression of browning genes in eWAT. Furthermore, the cell sizes and weight of the liver in the PGE-treated mice were significantly lower than those in the HFD group owing to the decreased levels of hepatic lipogenic mRNA expression (*FAS*, *SCD1*, *HMGCR*, and *ACAT*), some enzyme activities (G6PD, FAS, ME, and PAP), and the major transcriptional regulators, SREBP1 and SREBP2. Besides, the PGE treatment enhanced β-oxidation enzyme activity by increasing the expression of *PGC1*α mRNA, which in turn induced a significant decrease in hepatic LD formation and accumulation. In particular, the excessive release of FAs form dysfunctional and insulin-resistant adipocytes results in lipotoxicity, causing the accumulation of TG-derived toxic metabolites in liver [24,25]. PGE significantly reduced the plasma FFA level with a simultaneous increase in adipocyte FA uptake CD36 protein expression, leading to the reduction of hepatic lipotoxicity via the increased FFA flux to the liver.

In addition, increased accumulation of adipose tissue is accompanied by chronic adipose inflammation, which has been proposed to have an important role in the pathogenesis of obesity-related insulin resistance [4]. PGE significantly increased the adiponectin level, with a simultaneous decrease in the levels of leptin and TNF-α in adipocytes, which enhanced insulin sensitivity. Consistent with these findings, the PGE supplement reduced circulating levels of two inflammatory markers, TNF-α and resistin. Resistin is elevated in obesity and insulin resistance, and its deficiency in the mice led to improved glucose homeostasis. The PGE supplementation led to the normalization of plasma glucose and insulin levels, reflecting improved hepatic insulin sensitivity as evidenced by the reduced HOMA-IR and by the IPGTT data. In addition, PGE significantly decreased activities of hepatic glucose-regulating enzymes (PEPCK and G6Pase).

In recent years, studies have demonstrated that platycosides from the PG showed anti-obesitic, hypolipidemic and anti-diabetic properties that occurred via enhanced insulin sensitivity through decrease of plasma TC, glucose and insulin levels as well as hepatic cholesterol and TG in obese rodents [9,10,11]. This could probably be due to the difference in PGE preparation and composition. Moreover, suppression of body weight gain, body fat reduction, and improvement in insulin sensitivity by PGE supplementation were more potent in PGE in our study. These observations indicated that PGE has the potential to regulate glucose metabolism and hepatic lipid metabolism, thereby ameliorating hepatic steatosis and hyperglycemia in DIO mice. Importantly, an increase in WAT thermogenesis or browning of WAT can be accompanied by improved glucose homeostasis in vivo [26], similar to the effect of the PGE treatment.

## 5. Conclusions

**Figure 4:**
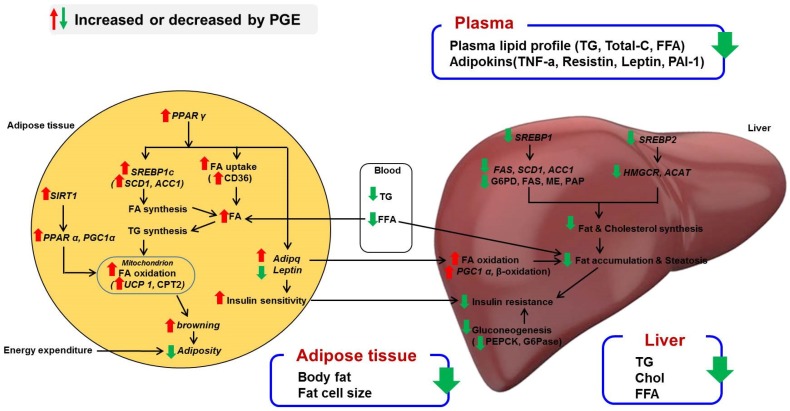
Proposed mechanism for PGE regarding anti-obesity effects. Schematic representation of the role of PGE in amelioration of obesity. PGE can contribute to browning of WAT in diet-induced obese mice through increasing fatty acid oxidation in mitochondria, which promotes energy expenditure. In addition, PPARγ activation by PGE controls adipokines, specifically the insulin-sensitizing hormones adiponectin and leptin. In the liver, PGE downregulates the mRNA expression of lipogenesis and cholesterol synthesis transcription factors, SREBP1 and SREBP2, thereby reducing hepatic steatosis and insulin resistance. PPARγ, peroxisome proliferator-activated receptor γ; SREBP1a, -1c, and -2, sterol regulatory element-binding proteins 1a, 1c, and 2; FAS, fatty acid synthase; ACC, acetyl-CoA carboxylase; SCD1, stearoyl-CoA desaturase; HMGCR, 3-hydroxy-3-methylglutaryl-CoA reductase; ACAT, acetyl-CoA acetyltransferase; *SIRT1*, sirtuin 1; *PPAR*α, peroxisome proliferator-activated receptor α; *PGC1*α, peroxisome proliferator-activated receptor gamma coactivator 1-alpha; *UCP1*, uncoupling protein 1; TNF-α, tumor necrosis factor-α; CPT2, carnitine palmitoyltransferase 2.

This study demonstrated that the PGE treatment normalized the body weight and body fat mass in the HFD-fed mice by increasing FAO. This partly changed the levels of thermogenesis-related genes such as *UCP1* as well as *PPAR*α, *PGC1*α, and *SIRT1* and thus could promote BAT-like features in WAT, as well as enhance energy expenditure. Furthermore, reducing the WAT mass by the PGE treatment enhanced the susceptibility to developing hepatic steatosis and insulin resistance. Figure 4 illustrates the possible mechanisms of the PGE effects for antiobesity. Taken together, PGE can suppress diet-induced obesity and modulate obesity-associated metabolic disorders. 

## Supplementary Materials

The following are available online at http://www.mdpi.com/2072-6643/8/9/532/s1, Table S1: Compounds of ethanol extracts of *Platycodon grandiflorum*.

## Abbreviations

ACAT	acetyl-CoA acetyltransferase
ACC	acetyl-CoA carboxylase
BAT	brown adipose tissue
FAS	fatty acid synthase
HFD	high-fat diet
HMGCR	3-hydroxy-3-methylglutaryl-CoA reductase
PPARγ	peroxisome proliferator-activated receptor gamma
*PGC1*α	PPARγ coactivator-1α
PGE	*Platycodon grandiflorus* root ethanol extract
SCD1	stearoyl-CoA desaturase1
SIRT1	sirtuin1
SREBP	sterol regulatory element-binding proteins
TNF-α	tumor necrosis factor alpha
*UCP1*	uncoupling protein 1
WAT	white adipose tissue
eWAT	epididydimal white adipose tissue

## References

[1] Akagiri S., Naito Y., Ichikawa H., Mizushima K., Takagi T., Handa O., Kokura S., Yoshikawa T. (2008). Bofutsushosan, an oriental herbal medicine, attenuates the weight gain of white adipose tissue and the increased size of adipocytes associated with the increase in their expression of uncoupling protein 1 in high-fat diet-fed male KK/Ta mice. J. Clin. Biochem. Nutr..

[2] Liu Z., Patil I.Y., Jiang T., Sancheti H., Walsh J.P., Stiles B.L., Yin F., Cadenas E. (2015). High-fat diet induces hepatic insulin resistance and impairment of synaptic plasticity. PLoS ONE.

[3] Chalkiadaki A., Guarente L. (2012). High-fat diet triggers inflammation-induced cleavage of SIRT1 in adipose tissue to promote metabolic dysfunction. Cell. Metab..

[4] Kim Y.-J., Choi M.-S., Cha B.Y., Woo J.T., Park Y.B., Kim S.R., Jung U.J. (2013). Long-term supplementation of honokiol and magnolol ameliorates body fat accumulation, insulin resistance, and adipose inflammation in high-fat fed mice. Mol. Nutr. Food Res..

[5] Jung U.J., Choi M.-S. (2014). Obesity and its metabolic complications: The role of adipokines and the relationship between obesity, inflammation, insulin resistance, dyslipidemia and nonalcoholic fatty liver disease. Int. J. Mol. Sci..

[6] Kwon E.-Y., Jung U.J., Park T., Yun J.W., Choi M.-S. (2015). Luteolin attenuates hepatic steatosis and insulin resistance through the interplay between the liver and adipose tissue in diet-induced obese mice. Diatetes.

[7] Lagouge M., Argmann C., Gerhart-Hines Z., Meziane H., Lerin C., Daussin F., Messadeq N., Milne J., Lambert P., Elliott P. (2006). Resveratrol improves mitochondrial function and protects against metabolic disease by activating SIRT1 and PGC-1a. Cell.

[8] Wang L., Teng R., Di L., Rogers H., Wu H., Kopp J.B., Noguchi C.T. (2013). PPARα and Sirt1 Mediate Erythropoietin Action in Increasing Metabolic Activity and Browning of White Adipocytes to Protect against Obesity and Metabolic Disorders. Diabetes.

[9] Lee J.-S., Choi M.-S., Seo K.-I., Lee H.I., Lee J.H., Kim M.J., Lee M.K. (2014). Platycodi radix saponin inhibits α-glucosidase in vitro and modulates hepatic glucose-regulating enzyme activities in C57BL/KsJ -*db*/*db* mice. Arch. Pharm. Res..

[10] Qin H., Du X., Zhang Y., Wang R. (2014). Platycodin D, a triterpenoid saponin from *Platycodon grandiflorum*, induces G_2_/M arrest and apoptosis in human hepatoma HepG_2_ cells by modulating the PI_3_K/Akt pathway. Tumour Biol..

[11] Zhang L., Wang Y., Yang D., Zhang C., Zhang N., Li M., Liu Y. (2015). Platycodon grandiflorus—An ethnopharmacological, phytochemical and pharmacological review. J. Ethnopharmacol..

[12] Collins S., Martin T.L., Surwit R.S., Robidoux J. (2004). Genetic vulnerability to diet-induced obesity in the C57BL/6J mouse: Physiological and molecular characteristics. Physiol. Behav..

[13] Shen W., Wang Y., Lu S.F., Hong H., Fu S., He S., Li Q., Yue J., Xu B., Zhu B.M. (2014). Acupuncture promotes white adipose tissue browning by inducing UCP1 expression on DIO mice. BMC Complement. Altern. Med..

[14] Vergnes L., Reue K. (2014). Adaptive Thermogenesis in White Adipose Tissue: Is Lactate the New Brown (ing)?. Diabetes.

[15] Cariou B., Charbonnel B., Staels B. (2012). Thiazolidinediones and PPARγ agonists: Time for a reassessment. Trends. Endocrinol. Metab..

[16] Rodgers J.T., Lerin C., Haas W., Gygi S.P., Spiegelman B.M., Puigserver P. (2005). Nutrient control of glucose homeostasis through a complex of PGC-1 alpha and SIRT1. Nature.

[17] Wu Z., Puigserver P., Andersson U., Zhang C., Adelmant G., Mootha V., Troy A., Cinti S., Lowell B., Scarpulla R.C. (1999). Mechanisms controlling mitochondrial biogenesis and respiration through the thermogenic coactivator PGC-1. Cell.

[18] Qian S.-W., Tang Y., Li X., Liu Y., Zhang Y.Y., Huang H.Y., Xue R.D., Yu H.Y., Guo L., Gao H.D. (2013). BMP4-mediated brown fat-like changes in white adipose tissue alter glucose and energy homeostasis. Proc. Natl. Acad. Sci. USA.

[19] Rachid T.L., Penna-de-Carvalho A., Bringhenti I., Aguila M.B., Mandarim-de-Lacerda C.A., Souza-Mello V. (2015). Fenofibrate (PPAR alpha agonist) induces beige cell formation in subcutaneous white adipose tissue from diet-induced male obese mice. Mol. Cell. Endocrinol..

[20] Samuel V.T., Petersen K.F., Shulman G.I. (2010). Lipid-induced insulin resistance: Unravelling the mechanism. Lancet.

[21] Tsuchida A., Yamauchi T., Takekawa S., Hada Y., Ito Y., Maki T., Kadowaki T. (2005). Peroxisome proliferator-activated receptor (PPAR) alpha activation increases adiponectin receptors and reduces obesity-related inflammation in adipose tissue: Comparison of activation of PPARalpha, PPARgamma, and their combination. Diabetes.

[22] Patel T.P., Soni S., Parikh P., Gosai J., Chruvattil R., Gupta S. (2013). Swertiamarin: An active lead from *Enicostemma littorale* regulates hepatic and adipose tissue gene expression by targeting PPAR-γ and improves insulin sensitivity in experimental NIDDM rat model. Evid. Based Complement. Altern. Med..

[23] Yamauchi T., Kamon J., Waki H., Terauchi Y., Kubota N., Hara K., Mori Y., Ide T., Murakami K., Tsuboyama-Kasaoka N. (2001). The fat-derived hormone adiponectin reverses insulin resistance associated with both lipoatrophy and obesity. Nat. Med..

[24] Kabir M., Catalano K.J., Ananthnarayan S., Kim S.P., Van Citters G.W., Dea M.K., Bergman R.N. (2005). Molecular evidence supporting the portal theory: A causative link between visceral adiposity and hepatic insulin resistance. Am. J. Physiol. Endocrinol. Metab..

[25] Lomonaco R., Ortiz-Lopez C., Orsak B., Webb A., Hardies J., Darland C., Finch J., Gastaldelli A., Harrison S., Tio F. (2012). Effect of adipose tissue insulin resistance on metabolic parameters and liver histology in obese patients with nonalcoholic fatty liver disease. Hepatology.

[26] Boström P., Wu J., Jedrychowski M.P., Korde A., Ye L., Lo J.C., Rasbach K.A., Boström E.A., Choi J.H., Long J.Z. (2012). A PGC1-α-dependent myokine that drives brown-fat-like development of white fat and thermogenesis. Nature.

