# Supplementary material for: Platycodon grandiflorus Root Extract Attenuates Body Fat Mass, Hepatic Steatosis and Insulin Resistance through the Interplay between the Liver and Adipose Tissue
Source: Nutrients. 2016 Aug 30;8(9):532. doi: 10.3390/nu8090532 (PMC5037519; doi:10.3390/nu8090532)
Supplement: Supplementary file 1 [file nutrients-08-00532-s001.docx]

Supplementary Materials: *Platycodon grandiflorus* Root Extract Attenuates Body Fat Mass, Hepatic Steatosis and Insulin Resistance through the Interplay between the Liver and Adipose Tissue

Ye Jin Kim, Ji-Young Choi, Ri Ryu, Jeonghyeon Lee, Su-Jung Cho, Eun-Young Kwon,
Mi-Kyung Lee, Kwang-Hyeon Liu, Yu Rina, Mi-Kyung Sung and Myung-Sook Choi

1. Materials and Methods

1.1. Energy Expenditure

Energy expenditure was measured using an indirect calorimeter (Oxylet; Panlab, Cornella, Spain). The mice were placed into individual metabolic chambers at 25 °C, with free access to
food and water. O_2_ and CO_2_ analyzers were calibrated with highly purified gas standards.
Oxygen consumption (*Vo_2_*) and carbon dioxide production (*V*co_2_) were recorded at 3-min intervals using a computer-assisted data acquisition program (Chart 5.2; AD Instrument, Sydney, Australia) over a 24-h period, and the data were averaged for each mouse. Energy expenditure (EE) was calculated according to the following formula: EE (kcal*/*day*/*kg of body weight^0^*^.^*^75^) = *V*o_2_ × 1*.*44 × (3*.*815 + (1*.*232 × *V*o_2_*/V*co_2_)).

1.2. Morphology of the Liver and Fat Tissues

The liver and epididymal adipose tissue (eWAT) were removed from each mouse. Samples were subsequently fixed in 10% (*v*/*v*) paraformaldehyde/phosphate-buffered saline and embedded in paraffin for staining with hematoxylin and eosin. Stained areas were visualized using a microscope set at 200× magnification.

1.3. Plasma Biomarkers

Plasma lipid concentrations were determined with commercially available kits. Plasma free fatty acid levels were measured using the Wako enzymatic kit (Wako Chemicals, Richmond, VA, USA), and triglyceride, total cholesterol, and HDL-cholesterol levels were determined using Asan enzymatic kits (Asan, Seoul, Korea). Plasma insulin, adipokines (resistin and leptin), and tumor necrosis factor alpha (TNF-α) were determined with a multiplex detection kit from Bio-Rad (Hercules, CA, USA). All samples were assayed in duplicate and analyzed with a Luminex 200 Labmap system (Luminex, Austin, TX, USA). Data analyses were done with the Bio-Plex Manager software version 4.1.1 (Bio-Rad, Richmond, CA, USA).

1.4. Fasting Blood Glucose, Intraperitoneal Glucose Tolerance Test, and Homeostatic Index of Insulin Resistance

The blood glucose concentration was measured by the glucose oxidase method using a glucose analyzer (Glucocard, Arkray, Japan) in whole blood obtained from the tail vein after food withholding for 12 h. The intraperitoneal glucose tolerance test (IPGTT) was performed at week 11. After 12 h of fasting, the mice were injected intraperitoneally with glucose (0.5 g/kg of body weight). The blood glucose level was determined from the tail vein at 0, 30, 60, and 120 min after the glucose injection. The homeostatic index of insulin resistance (HOMA-IR) was calculated according to the homeostasis assessment model as follows: HOMA-IR = (fasting glucose (mmol/L) × fasting insulin (IU*/*mL))*/*22*.*51.

1.5. Hepatic Lipid Content

Hepatic lipids were extracted as previously described [1], and then dried lipid residues were dissolved in 1 mL of ethanol for triglyceride, cholesterol, and free fatty acid (FFA) assays. Triton X-100 and a sodium cholate solution in distilled water were added to 200 μL of a dissolved lipid solution for emulsification. Hepatic triglyceride, cholesterol, and FFA contents were analyzed with the same enzymatic kits that were used for the plasma analysis.

1.6. Glucose- and Lipid-Regulating Enzyme Activity

To measure glucose- and lipid-regulating enzyme activities in the liver, hepatic cytosolic, mitochondrial, and microsomal preparations were obtained according to Shao et al. [2], with a slight modification, and protein concentrations were determined using the Bradford method [3].
Glucose-6-phosphate dehydrogenase (G6PD) [4], fatty acid synthase (FAS) [5], malic enzyme (ME) [6], and phosphatidate phosphohydrolase (PAP) [7] activities were measured as previously described. Glucose-6-phosphatase (G6Pase) activity was determined using the method of Alegre et al. [8]. Phosphoenolpyruvate carboxykinase (PEPCK) activity was monitored in the direction of oxaloacetate synthesis using a spectrophotometric assay developed by Bentle and Lardy [9]. Fatty acid β-oxidation was measured spectrophotometrically by monitoring the reduction of NAD to NADH in the presence of palmitoyl-CoA as described by Lazarow [5], with a slight modification.

1.7. Analysis of Gene Expression

The eWAT and liver were homogenized in the TRIzol reagent (Invitrogen, Grand Island, NY, USA), and total RNA was isolated according to the manufacturer’s instructions. The total RNA was converted to cDNA using the QuantiTect Reverse Transcription kit (Qiagen Gmbh, Hilden, Germany). mRNA expression was quantified by quantitative real-time polymerase chain reaction (PCR) using the QuantiTect SYBR Green PCR kit (Qiagen) and SDS7000 sequence detection system (Applied Biosystems, CA, USA). Each cDNA sample was amplified using primers for the glyceraldehyde-3-phosphate dehydrogenase (*GAPDH*) gene) labeled with SYBR green dye.
The amplification was performed as follows: 10 min at 90 °C, 15 s at 95 °C, and 60 s at 60 °C for a total of 40 cycles. The cycle threshold (Ct) was defined as the cycle at which a statistically significant increase in the SYBR green emission intensity occurred. The Ct data were normalized relative to those for the housekeeping gene, *GAPDH*, which is stably expressed in mice. Relative gene expression was calculated with the 2^∆∆Ct^ method [10].

1.8. Primer

The primer were designed using a Primer 5.0 software (Primer-E Ltd., Plymouth, UK), PPARγ (Forward: 5′-GAG TGT GAC GAC AAG ATT TG-3′, Reverse: 5′-GGT GGG CCA GAA TGG CAT CT-3′), SREBP1a (Forward: 5′-TAG TCC GAA GCC GGG TGG GCG CCG GCG CCA T-3′, Reverse: 5′-GAT GTC GTT CAA AAC CGC TGT GTG TCC AGT TC-3’), SREBP1c (Forward: 5′-GGA GCC ATG GAT TGC ACA TT-3′, Reverse: 5′-CCT GTC TCA CCC CCA GCA TA-3′), SREBP2 (Forward: 5′-CAC AAT ATC ATT GAA AAG CGC TAC CGG TCC-3′, Reverse: 5′-TTT TTC TGA TTG GCC AGC TTC AGC ACC ATG-3′), SCD1 (Forward: 5′-CCC CTG CGG ATC TTC CTT AT-3′, Reverse: 5′-AGG GTC GGC GTG TGT TTC T-3′), FAS (Forward: 5′-GCT GCG GAA ACT TCA GGA AAT-3′, Reverse: 5′-AGAGAC GTG TCA CTC CTG GAC TT-3′), ACC (Forward: 5′-GGA CAG ACT GAT CGC AGA GAA AG-3′, Reverse: 5′-TGG AGA GCC CCA CAC ACA-3′), HMGCR (Forward: 5′-TTC ACG CTC ATA GTC GCT GGA TAG-3′, Reverse: 5′-TGG TTC AAT TCT CTT GGA CAC ATC TTC-3′), ACAT (Forward: 5′-CTC ACG GCA GGA ACA GGA TAC G-3′, Reverse: 5′-TTC TTC ATC TTC TTT CAC CAC CAC ATC-3′), SIRT1 (Forward: 5′-TGT GAA GTT ACT GCA GGA GTG TAA-3′, Reverse: 5′-GCA TAG ATA CCG TCT CTT GAT CTG AA-3’), PPARα (Forward: 5′-GGC ACC CTC ACA TCA TCA AAC TG-3′, Reverse: 5′-TGG AAC AGA CGG CGG CTT TC-3′), PGC1 α (Forward: 5′-AAG TGT GGA ACT CTC TGG AAC TG-3′, Reverse: 5′-GGG TTA TCT TGG TTG GCT TTA TG-3’), UCP1 (Forward: 5′-AGA TCT TCT CAG CCG GAG TTT-3′, Reverse: 5′-CTG TAC AGT TTC GGC AAT CCT-3’), Adipoq (Forward: 5′-GGT CTT CTT GGT CCT AAG GGT GAG-3′, Reverse: 5′-GCG GCT TCT CCA GGC TCT C-3′), Leptin (Forward: 5′-CTC CAA GGT TGT CCA GGG TT-3’, Reverse: 5′-AAA ACT CCC CAC AGA ATG GG-3′), TNFα (Forward: 5′-GCA GGT CTA CTT TAG AGT CAT TGC-3’, Reverse: 5′-TCC CTT TGC AGA ACT CAG GAA TGG-3′), GAPDH (Forward: 5′-ACA ATG AAT ACG GCT ACA GCA ACA G-3′, Reverse: 5′-GGT GGT CCA GGG TTT CTT ACT CC-3′).

1.9. Western Blot Analysis

Epididymal adipose tissue protein was extracted with lysis buffer and quantified using the Bradford method. Total protein (80–100 g) was electrophoresed on 10% SDS polyacrylamide gels and transferred to polyvinylidene fluoride membranes (Millipore), blocked, and probed with mouse anti-*PPAR*γ (1:1000; Santa Cruz Biotechnology, Santa Cruz, CA, USA), rabbit anti-CD36 (1:1000; Abcam), rabbit anti-PGC 1a (1:1000; Abcam), rabbit anti-CPT2 (1:1000; Abcam) and mouse anti-b-actin (1:1000; Cell Signaling), respectively. The immunoreactive antigen was then recognized with a horseradish peroxidase-labeled anti-rabbit or anti-mouse IgG (1:1000; Cell Signaling) and an enhanced ECL kit from Pierce Biotechnology (Rockford, IL, USA). The immunoreactive bands were quantified with a G-box (50S; BI System, MD, USA).

1.10. Statistical Analysis

Data were expressed as the mean ± standard error of the mean (SEM). Differences among the ND, HFD and PGE groups were assessed for significance using one-way analysis of variance
(one-way ANOVA), as calculated using SPSS software (SPSS Inc., Chicago, IL, USA). Any differences identified between groups at each time-point were analyzed further using Duncan’s multiple-range post-hoc test. Results were considered statistically significant at *p* < 0.05.

**Table S1.** Compounds of ethanol extracts of *Platycodon grandiflorum*.

| **No.** | **Saponin Name** | **Content (µg/mg)** | **Content (%)** |
| --- | --- | --- | --- |
| 1 | Deapioplatycoside E | 0.49 | 0.05 |
| 2 | Platycoside E | 0.56 | 0.06 |
| 3 | Deapioplatycodin D3 | 0.51 | 0.05 |
| 4 | Platycodin D3 | 1.87 | 0.19 |
| 5 | Platyconic acid B Lactone | 0.01 | 0.00 |
| 6 | Polygalacin D3 | 0.56 | 0.06 |
| 7 | Platycoinc acid A | 0.88 | 0.09 |
| 8 | 3″-*O*-acetylplatyconic acid A | 3.50 | 0.35 |
| 9 | Platycodin D2 | 1.84 | 0.18 |
| 10 | Platycodin D | 1.71 | 0.17 |
| 11 | 3″-*O*-acetylplatycodin D2 | 1.56 | 0.16 |
| 12 | Polygalacin D2 | 0.59 | 0.06 |
| 13 | Polygalacin D | 0.27 | 0.03 |
| 14 | 3″-*O*-acetylplatycodin D | 10.25 | 1.03 |
| 15 | Platycodin V | 1.61 | 0.16 |
| 16 | Platycodin A | 10.28 | 1.03 |
| 17 | 2″-*O*-acetylpolygalacin D2 | 2.34 | 0.23 |
| 18 | 2″-*O*-acetylpolygalacin D | 1.28 | 0.13 |

Analysis of platycosides by LC-MS/MS, LC-MS/MS analysis was performed on a Nexera2 LC system (Shimadzu Corporation, Kyoto, Japan) connected to a triple quadrupole mass spectrometer (LC-MS 8040; Shimadzu) equipped with an electrospray ionization (ESI) source in the negative mode. The chromagraphic separation was carried out on a Kinetex C18 column (100 × 2.1 mm, 2.6 μm, Phenomenex, Torrance, CA, USA). Eluent A consisted of 0.1% formic acid in H_2_O, and eluent B consisted of 0.1% formic acid in acetonitrile. To achieve the best resolution of the platycosides, gradient elution was conducted as follows: 5% B (0–2 min), 20% B (2–5 min), 20% B (5–10 min), 30% B (10–17 min), 30% B (17–30 min), and 5% B (30–35 min). The electrospray ionization (ESI) mass spectrometer was operated in the positive mode. ESI source settings were as follows: capillary voltage −3000 V, vaporizer temperature 300 °C, capillary temperature 350 °C, sheath (Neb) gas 3 L/min, ion sweep gas 2.0 Arb, Aux gas 10 Arb, and drying gas 8 L/min.

Reference

1. Folch, J.; Lees, M.; Sloan-Stanley, G.H. A simple method for isolation and purification of total lipids from animal tissues. *J. Biol. Chem.* **1957**, *226*, 497–409.
2. Shao, R.; Ring, S.C.; Tarloff, J.B. Coincubation of rat renal proximal tubules with hepatic subcellular fractions potentiates the effects of para-aminophenol. *Fundam. Appl. Toxicol*. **1997**, *39*, 101–108.
3. Bradford, M.M. A rapid and sensitive method for the quantitation of microgram quantities of protein utilizing the principle of protein-dye binding. *Anal. Biochem*. **1976**, *72*, 248–254.
4. Rudack, D.; Chisholm, E.M.; Holten, D. Rat liver glucose 6-phosphate dehydrogenase. Regulation by carbohydrate diet and insulin. *J. Biol. Chem.* **1971**, *246*, 1249–1254.
5. Lazarow, P.B. Assay of peroxisomal beta-oxidation of fatty acids. *Methods Enzymol*. **1981**, *72*, 315–319.
6. Ochoa, S. Malic enzyme: Malic enzymes from pigeon and wheat germ. In *Methods in Enzymology*;
   Colowick, S.P., Kaplan, N.O., Eds.; Academic Press: New York, NY, USA, 1955; Volume 1, pp. 323–326.
7. Walton, P.A.; Possmayer, F. Mg2-dependent phosphatidate phosphohydrolase of rat lung: Development of an assay employing a defined chemical substrate which reflects the phosphohydrolase activity measured using membrane-bound substrate. *Anal. Biochem.* **1985**, *151*, 479–486.
8. Alegre, M.; Ciudad, C.J.; Fillat, C.; Guinovart, J.J. Determination of glucose-6-phosphatase activity using the glucose dehydrogenase-coupled reaction. *Anal. Biochem*. **1988**, *173*, 185–189.
9. Bentle, L.A.; Lardy, H.A. Interaction of anions and divalent metal ions with phosphoenolpyruvate carboxykinase. *J. Biol. Chem.* **1976**, *251*, 2916–2921.
10. Schmittgen, T.D.; Livak, K.J. Analyzing real-time PCR data by the comparative C(T) method. *Nat. Protoc*. **2008**, *3*, 1101–1108.
